# Reproducibility of Serologic Assays for Influenza Virus A (H5N1)

**DOI:** 10.3201/eid1508.081754

**Published:** 2009-08

**Authors:** Iain Stephenson, Alan Heath, Diane Major, Robert W. Newman, Katja Hoschler, Wang Junzi, Jacqueline M. Katz, Jerry P. Weir, Maria C. Zambon, John M. Wood

**Affiliations:** University of Leicester, Leicester, UK (I. Stephenson); National Institute for Biological Standards and Controls, Potters Bar, UK (A. Heath, D. Major, R.W. Newman, J.M. Wood); Health Protection Agency, Colindale, UK (K. Hoschler, M.C. Zambon); Centers for Disease Control and Prevention, Atlanta, Georgia, USA (J.M. Katz); Food and Drug Administration, Rockville, Maryland, USA (J.P. Weir); National Institute for the Control of Pharmaceutical and Biological Products, Beijing, People’s Republic of China (W. Junzhi)

**Keywords:** pandemic, vaccine, immunogenicity, influenza, viruses, serologic assays, expedited, research

## Abstract

Hemagglutination-inhibition (HI) and neutralization are used to evaluate vaccines against influenza virus A (H5N1); however, poor standardization leads to interlaboratory variation of results. A candidate antibody standard (07/150) was prepared from pooled plasma of persons given clade 1 A/Vietnam/1194/2004 vaccine. To test human and sheep antiserum, 15 laboratories used HI and neutralization and reassortant A/Vietnam/1194/2004, A/turkey/Turkey/1/2005 (clade 2.2), and A/Anhui/1/2005 (clade 2.3.4) viruses. Interlaboratory variation was observed for both assays, but when titers were expressed relative to 07/150, overall percentage geometric coefficient of variation for A/Vietnam/1194/2004 was reduced from 125% to 61% for HI and from 183% to 81% for neutralization. Lack of reduced variability to clade 2 antigens suggested the need for clade-specific standards. Sheep antiserum as a standard did not reliably reduce variability. The World Health Organization has established 07/150 as an international standard for antibody to clade 1 subtype H5 and has an assigned potency of 1,000 IU/ampoule.

## Abstract 2

Results for clade 1 viruses were more consistent among laboratories when a standard antibody was used.

Influenza viruses agglutinate erythrocytes by binding to cell surface sialic acid. Agglutination may be blocked by strain-specific antibody detectable in hemagglutination-inhibition (HI) tests (*1*)*.* Because serum HI titers correlate with protection (*2*)*,* they are used to evaluate immunogenicity of influenza vaccines (*3**–**5*)*.* However, conventional HI is generally insensitive for the detection of antibody to avian strains (*6**,**7*)*.* Alternative serologic assays, including neutralization and HI with horse erythrocytes (hHI), are used to evaluate vaccine for pandemics (*7**–**9*)*.* HI sensitivity for avian influenza increases when erythrocytes that express sialic acid containing α2,6-galactose linkages are used; these erythrocytes are preferentially recognized by avian hemagglutinin (*8**,**9*)*.* Virus neutralization can be developed for any influenza subtype, although use of live virus may require heightened biocontainment.

Variability of influenza serologic assay results is partly attributed to differences in protocols and expression of endpoints (*10**,**11*)*.* Assay variability limits comparison of candidate influenza virus subtype H5N1 vaccines in different clinical trials, posing challenges for licensure, particularly if specific seroprotective titers are required as endpoints (*3**–**5*)*.* The use of bioassay standards to improve interlaboratory agreement is well recognized (*12*,*13*). However, the antigenic diversity of subtype H5N1 viruses (*14*) may pose challenges in maintaining relevant strain-specific antibody standards. We assessed the reproducibility of neutralization and hHI tests for influenza virus A (H5N1) and evaluated the suitability of a standard (freeze-dried plasma pool, obtained from persons vaccinated with clade 1 subtype H5N1, called 07/150) for detection of antibody.

## Methods

### Serum Samples

We used 14 serum samples (coded A–N) from persons who had received nonadjuvanted or adjuvanted split-product vaccine derived from reassortant clade 1 virus (A/Vietnam/1194/2004 or A/Vietnam/1203/2004). Samples included 3 prevaccination and postvaccination paired samples and 4 postvaccination samples. Postvaccination samples were generally obtained within 42 days of vaccination. Samples A and L were identical duplicates from a person primed with clade 0 vaccine and boosted with adjuvanted clade 1 vaccine. Sample K was from a person known to show nonspecific assay reactivity for antibodies to H5. Sample M was pooled negative human serum. Sheep antiserum to influenza subtype H5N1 (samples O and P) was supplied by the US National Institute of Allergy and Infectious Diseases and the UK National Institute for Biologic Standards and Controls, respectively. Serum P was produced by intramuscular administration of 20 µg hemagglutinin (HA) from A/Vietnam/1194/NIBRG-14 with Freund complete adjuvant, followed by 3 more injections with 10 µg HA with Freund incomplete adjuvant. Serum was collected 5 weeks after the first vaccination. Serum O was produced in a similar manner, with bromelin-cleaved purified HA. A single animal was vaccinated with A/turkey/Wisconsin/68 H5 bromelin-cleaved HA and received booster vaccinations with purified HA from A/Vietnam/1203/2004.

### Candidate Standard 07/150

Standard 07/150 contained freeze-dried plasma from 9 persons who had received inactivated whole-virus A/Vietnam/1194/NIBRG-14 vaccine. Four donations (total volume 3 L) were obtained from Omninvest, Hungary (vaccine contained aluminum phosphate), and 5 donations (total volume 2 L) were obtained from Sinovac, People’s Republic of China (vaccine contained aluminum hydroxide). Persons gave informed consent after studies had received approval by appropriate ethics committees. Donations were negative for antibodies to HIV-1, HIV-2, hepatitis B surface antigen, and hepatitis C RNA. Plasma was pooled and freeze dried at the National Institute for Biologic Standards and Controls, according to standard procedures (*15*) to produce 1-mg ampoules and stored at –20°C. Stability studies found no significant change in titers after 8 months at –20°C, +4°C, or +20°C when compared with samples stored at –70°C.

### Virus Reagents

Reassortant subtype H5N1 influenza viruses were prepared by reverse genetics from wild-type viruses, amplified in 10-day-old embryonated hens’ eggs, and stored at –80°C. Each virus passed internationally approved safety testing (*16*), which permitted use at Biosafety Level 2–enhanced facilities. The National Institute for Biologic Standards and Controls supplied NIBRG-14 (A/Vietnam/1194/2004, clade 1) and NIBRG-23 (A/turkey/Turkey/1/2005, clade 2.2) viruses, and the Centers for Disease Control and Prevention supplied IBCDC-RG5 (A/Anhui/1/2005, clade 2.3.4).

### Study Design

Fifteen laboratories from 9 countries agreed to participate and were assigned a code from 1 to 15. One additional laboratory returned titers from 1 neutralization and 1 pseudotype assay and was excluded from analysis.

The participating laboratories were sent reagents on solid CO_2_, asked to store serum at –20°C and viruses at –70°C, and instructed to reconstitute 07/150 with 1 mL distilled water and to test it and the serum for antibodies to each antigen, using hHI and neutralization, on at least 3 separate occasions. Suggested protocols were supplied, but participating laboratories could use in-house assays.

### Statistical Analyses

Neutralization and hHI data consisted of replicate absolute titers, expressed as the reciprocal of serum dilution, and represented the last dilution giving a positive response from a doubling-dilution series. If the initial dilution did not give a positive response, the titer was recorded as less than the reciprocal initial dilution, e.g., <10 if the starting dilution was 1:10. Serum was interpreted as negative if no titer was detected and positive if any titer was detected. For calculation, negative titers were assigned the value of half the minimum detectable titer, and titers greater than the final dilution were assigned a value 2× the largest titer. These values represent the hypothetical adjacent dilution steps in the doubling-dilution series. This convention enables comparison of overall mean titers among groups on a consistent basis.

We calculated the geometric mean titer (GMT) for each serum, virus, and assay combination. Overall titers were calculated as the GMT of the individual laboratory means. Interlaboratory variation was expressed as percentage geometric coefficient of variation (%GCV) between the individual laboratory GMTs. The distribution of hHI or neutralization titers does not represent a continuous variable, and the results from using different viruses within laboratories are not independent. Thus, use of parametric modeling techniques, such as analysis of variance, to characterize intra- and interlaboratory variability was precluded.

To assess intralaboratory variation, we calculated the percentage of endpoints of replicate tests for identical serum samples A and L that differed >2-fold or >4-fold for each antigen and assay in each laboratory. We also compared the percentage of replicate tests returned for all serum samples and postvaccination samples that differed by >2-fold or >4-fold for each antigen and assay.

To assess interlaboratory variation, we compared differences between hHI and neutralization GMTs for 07/150 by different laboratories by using a paired nonparametric Wilcoxon signed-rank test for each antigen separately. For each laboratory, the difference in GMT between hHI and neutralization for NIBRG-14 was calculated, and these differences were compared with zero by using the Wilcoxon signed-rank test. Similarily, the results for hHI with each antigen were compared, taking the laboratory differences between the hHI GMT for NIBRG-14 and NIBRG-23 and the differences between NIBRG-14 and IBCDC-RG5 and comparing these differences with zero. The same was done for neutralization assays. We also compared differences among overall (for all laboratories) mean GMT for all serum samples by using a paired nonparametric Wilcoxon signed-rank test for each antigen separately; e.g., for NIBRG-14, the difference between the overall mean GMT for hHI and neutralization was calculated for each sample, and these differences were compared with zero. The nonparametric tests use the ranks of observed titers to calculate the significance of differences among groups and are unaffected by the value chosen to represent titers below the initial dilution or greater than the highest dilution used in the individual assays.

To assess a standard’s ability to improve interlaboratory agreement, we expressed titers relative to 07/150 by taking the ratio of the GMT for a sample to the GMT for 07/150 and multiplying it by an assigned value for 07/150. The assigned value was the overall GMT by hHI and neutralization. The effect on interlaboratory agreement and %GCV is independent of the value chosen.

To evaluate improvement in interlaboratory agreement from expressing titers relative to 07/150 (or sheep antiserum), we calculated %GCV between laboratory GMTs, both absolute and relative, for each sample. We then calculated the difference between the %GCV of the laboratory GMT of absolute titers and the %GCV of the laboratory GMT of relative titers. Using the Wilcoxon signed-rank test for each antigen separately, we compared these differences with zero.

## Results

### Assays

All participating laboratories returned at least 3 replicates by both assays, except laboratory 11, which did not perform hHI. Negative serum M was excluded because all titers were negative, except in laboratory 3, which reported 1 positive ( titer 45) and 2 negative neutralization titers.

### Intralaboratory Reproducibility: Identical Samples A and L

The numbers of intralaboratory comparisons of samples A and L differing by >2-fold were 1 (2.4%), 2 (4.8%), and 1 (2.4%) of 42 for NIBRG-14, NIBRG-23, and IBCDC-RG5, respectively, by hHI compared with 3 (7.1%), 3 (7.1%), and 1 (2.4%) of 42 by neutralization. Overall, 4 of 126 (3.1%) comparisons of identical samples by hHI differed by >2-fold compared with 7 of 127 (5.5%) by neutralization. No samples differed by >4-fold by either assay.

### Intralaboratory Reproducibility: Replicate Assays

**Figure 1:**
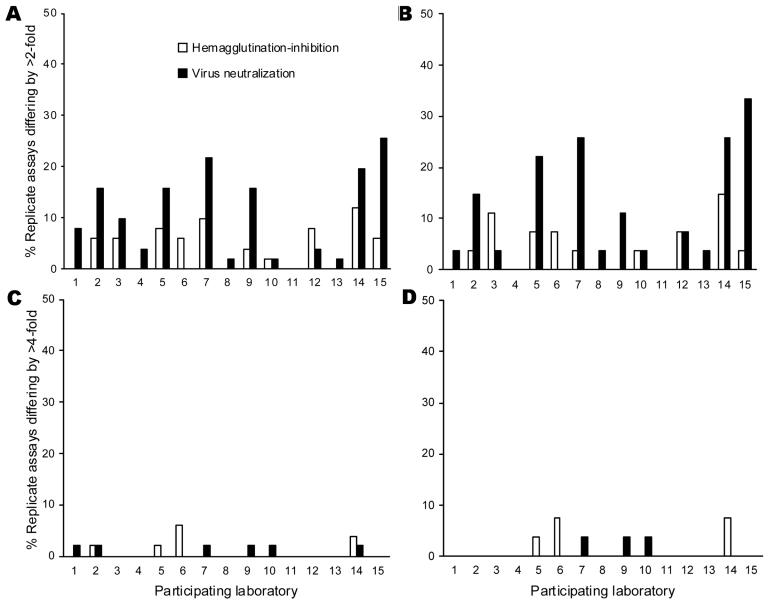
Intralaboratory reproducibility showing proportion (%) of replicate assays differing by >2-fold (A, B) and >4-fold (C, D) by horse hemagglutination-inhibition and neutralization assays for each participating laboratory for all serum samples (A, C) and postvaccination serum samples (B, D). Laboratory 11 did not return hemagglutinin-inhibition result.

The proportion of serum samples for which replicate assays differed by >2-fold and >4-fold was assessed in each laboratory for all serum and postvaccination serum samples (Figure 1). By hHI, 13 of 14 (93%) laboratories reported >90% replicate titers within a 2-fold range. By neutralization, intralaboratory reproducibility was more variable; 9 of 15 (60%) laboratories reported >90% replicate titers within a 2-fold range. For postvaccination serum, greater variability was found with neutralization; laboratories 7, 14, and 15 reported >25% replicates differing by >2-fold. Three laboratories reported 3.7%–7.4% of replicate samples differing by >4-fold by either assay.

### Interlaboratory Reproducibility of Absolute Titers: 07/150

All laboratories reported positive titers to 07/150, but variation was marked (Table 1). By hHI, the GMTs, ranges, and %GCVs in all laboratories to NIBRG-14, NIBRG-23, and IBCDC-RG5 were 140 (25–406; 16-fold difference, 112%), 102 (25–320; 13-fold difference, 109%), and 91 (25–256; 10-fold difference, 101%), respectively. By neutralization, the GMTs, ranges, and %GCVs of titers to NIBRG-14, NIBRG-23, and IBCDC-RG5 were 518 (127–2032; 16-fold difference, 120%), 291 (52–1810; 35-fold difference, 140%), and 299 (80–806; 10–fold difference, 109%), respectively. Titers were higher with neutralization than with hHI (p = 0.003, NIBRG-14; p = 0.005, NIBRG-23; p = 0.005, IBCDC-RG5; Wilcoxon signed-rank test for all comparisons). The GMT for NIBRG-14 was higher than that for clade 2 viruses (hHI: p = 0.039, NIBRG-23; p = 0.028, IBCDC-RG5; neutralization: p = 0.004, NIBRG-23; p = 0.008, IBCDC-RG5; Wilcoxon signed-rank test).

**Table 1:** Geometric mean titers (reciprocal shown) and geometric coefficient of variation for the candidate antibody standard 07/150, measured by each participating laboratory for each influenza virus*

Laboratory no.	Assay tested and virus used (phylogenetic clade)						
	hHI†				Neutralization†		
	NIBRG-14‡§ clade 1	NIBRG-23‡ clade 2.2	IBCDC-RG5§ clade 2.3		NIBRG-14‡§ clade 1	NIBRG-23‡ clade 2.2	IBCDC-RG5§ clade 2.3
1	101	160	80		480	605	419
2	25	25	101		1016	640	806
3	403	320	160		1810	1810	226
4	160	80	80		336	233	469
5	406	256	256		450	270	632
6	80	160	202		320	320	202
7	101	63	63		127	101	80
8	160	80	80		320	160	160
9	160	183	115		160	52	80
10	160	80	105		960	210	733
11¶	ND	ND	ND		640	254	320
12	403	160	202		320	160	160
13	80	32	25		640	320	320
14	160	127	25		2032	905	508
15	101	63	63		731	313	471
Overall GMT	140	102	91		518	291	299
GCV, %	112	109	101		120	140	109

*GMT. geometric mean titer; GCV, geometric coefficient of variation; hHI, hemagglutination-inhibition assay using horse erythrocytes; neutralization, virus neutralization assay; ND, not done. †GMT neutralization vs. hHI (NIBRG-14, p = 0.003; NIBRG-23, p = 0.005; IBCDC-RG5, p = 0.005 by Wilcoxon signed-rank test). ‡GMT NIBRG-14 vs. NIBRG-23 (hHI, p = 0.039; neutralization, p = 0.004 by Wilcoxon signed-rank test). §GMT NIBRG-14 vs. IBCDC-RG5 (hHI, p = 0.028; neutralization, p = 0.008 by Wilcoxon signed-rank test). ¶Laboratory 11 did not return hHI data.

### Interlaboratory Reproducibility of Absolute Titers

GMT and %GCV for each serum sample, virus, and assay were calculated (Table 2). For postvaccination serum, both assays showed higher titers to NIBRG-14 than to clade 2 viruses. For all serum, neutralization gave higher titers than hHI (p = 0.001, NIBRG-14; p = 0.008, NIBRG-23; p = 0.001, IBCDC-RG5; Wilcoxon signed-rank test) and fewer negative values but displayed more range variation, particularly in prevaccination serum. Absolute titers for sheep serum samples O and P were highly variable; %GCV was 147%–582% for hHI and 117%–283% for neutralization.

**Table 2:** Geometric mean titers and percentage coefficient of variations of absolute titers and titers relative to candidate antibody standard 07/150 for each serum sample for each influenza virus by hHI and neutralization*†

Serum sample	Virus assay, antigen, and clade						
	hHI (GMT, %GCV, %GCV relative to 07/150)				Neutralization (GMT, %GCV, %GCV relative to 07/150)		
	NIBRG-14 clade 1	NIBRG-23 clade 2.2	IBCDC-RG5 clade 2.3		NIBRG-14 clade 1	NIBRG-23 clade 2.2	IBCDC-RG5 clade 2.3
Prevaccination							
B	7, 110, 72	7, 111, 86	6, 112, 85		12, 176, 202	12, 141, 173	10, 68, 223
N	6, 31, 46	5, 29, 18	5, 22, 0		14, 175, 228	13, 160, 215	10, 186, 250
J	10, 111, 123	8, 81, 155	6, 43, 96		19, 175, 228	18, 218, 309	11, 102, 287
Postvaccination							
Low							
C	34, 84, 36	17, 29, 18	10, 126, 164		63, 183, 80	27, 201, 236	23, 122, 206
D	15, 141, 105	8, 110, 127	6, 68, 45		19, 232, 248	15, 215, 288	12, 98, 226
High							
E	104, 133, 58	60, 96, 36	8, 128, 141		148, 191, 81	87, 159, 42	20, 118, 223
F	78, 97, 55	16, 141, 116	20, 104, 105		83, 144, 62	18, 152, 234	35, 147, 196
G	281, 152, 61	147, 102, 131	44, 163, 144		504, 132, 37	274, 217, 83	140, 103, 45
H	95, 118, 66	42, 144, 116	18, 106, 111		130, 166, 74	91, 157, 33	34, 127, 154
I	351, 125, 60	93, 91, 47	107, 75, 44		379, 199, 71	106, 207, 191	161, 136, 41
A	391, 138, 55	335, 114, 34	448, 119, 51		1,389, 86, 45	1,313,125, 76	2,893, 78, 63
L	391, 147, 61	398, 145, 42	480, 108, 44		1,453, 104, 63	1,520, 101, 85	3,097, 73, 52
False positive (K)	37, 120, 34	24, 136, 135	8, 81, 117		52, 185, 143	44, 185, 53	13, 112, 298
Sheep							
O	48, 262, 285	30, 245, 338	17, 147, 319		216, 139, 53	148, 170, 33	49, 117, 156
P	1,857, 582, 535	1,171, 496, 487	1,338, 487, 545		7,317, 148, 196	732, 283, 144	3,806, 145, 59

*GMT geometric mean titer; %GCV, percentage geometric coefficient of variation; hHI, hemagglutination-inhibition assay using horse erythrocytes; neutralization, virus neutralization assay. (Paired samples were B and C, N and F, and J and H.) †Overall serum samples, GMT by neutralization vs. hHI (p = 0.001, NIBRG-14; p = 0.008, NIBRG-23; p = 0.001, IBCDC-RG5; Wilcoxon signed-rank test).

When summarized over all serum samples, the best interlaboratory agreement was for IBCDC-RG5 by hHI and neutralization; %GCVs were 108% and 112%, respectively (Table 3). The worst interlaboratory agreement was for neutralization with NIBRG-23 and NIBRG-14; %GCVs were 185% and 183%, respectively.

**Table 3:** Interlaboratory geometric coefficients of variation of absolute titers and titers and relative to candidate antibody standard 07/150 and sheep serum summarized over all serum for each influenza virus tested by hHI and neutralization*

%GCV, virus strain	hHI, all serum samples, median (min–max)	Neutralization, all serum samples, median (min–max)
%GCV of absolute titer		
**A/Vietnam/1194/NIBRG-14 clade 1**	**125 (31–582)†**	**175 (86–232)†**
A/Turkey/23/NIBRG-23 clade 2.2	114 (29**–**496)	170 (101**–**283)
A/Anhui/IBCDC-RG5 clade 2.3	108 (22**–**487)	112 (68**–**147)
%GCV of titers relative to 07/150		
**A/Vietnam/1194/NIBRG-14 clade 1**	**61 (34–535)**	**77 (37–285)**
A/Turkey/23/NIBRG-23 clade 2.2	106 (18**–**487)	144 (33**–**309)
A/Anhui/IBCDC-RG5 clade 2.3	105 (0**–**545)	196 (41**–**298)
%GCV of titers relative to serum P		
**A/Vietnam/1194/NIBRG-14 clade 1**	**796 (39–1,020)**	**249 (162–381)**
A/Turkey/23/NIBRG-23 clade 2.2	689 (7**–**953)	237 (90**–**844)
A/Anhui/IBCDC-RG5 clade 2.3	752 (0**–**1,005)	195 (66**–**263)
**A/Vietnam/1194/NIBRG-14 clade 1 (excluding laboratory 5)**	**68 (32–174)**	**255 (162–396)**
A/Turkey/23/NIBRG-23 clade 2.2 (excluding laboratory 5)	70 (7**–**222)	237 (90**–**844)
A/Anhui/IBCDC-RG5 clade 2.3 (excluding laboratory 5)	51 (0**–**148)	195 (66**–**262)
%GCV of titers relative to serum 0		
**A/Vietnam/1194/NIBRG-14 clade 1**	**442 (21–725)**	**78 (41–213)**
A/Turkey/23/NIBRG-23 clade 2.2	373 (20**–**804)	111 (29**–**204)
A/Anhui/IBCDC-RG5 clade 2.3	306 (0**–**812)	199 (44**–**323)
**A/Vietnam/1194/NIBRG-14 clade 1 (excluding laboratories 5, 6, 12)**	**39 (24–91)**	**82 (34–225)**
A/Turkey/23/NIBRG-23 clade 2.2 (excluding laboratories 5, 6, 12)	113 (22**–**225)	97 (25**–**186)
A/Anhui/IBCDC-RG5 clade 2.3	100 (0**–**198)	194 (47**–**299)

*%GCV, geometric coefficient of variation; hHI, hemagglutination-inhibition assay using horse erythrocytes; neutralization, virus neutralization assay; min–max, minimum–maximum. **Boldface** indicates homologous clade 1 strain. †%GCV absolute titer vs. relative 07/150 for NIBRG-14 (^*^hHI p = 0.001, neutralization p = 0.002; Wilcoxon signed-rank test).

### Reproducibility of Relative Titers: 07/150 or Sheep Serum as Standard

**Figure 2:**
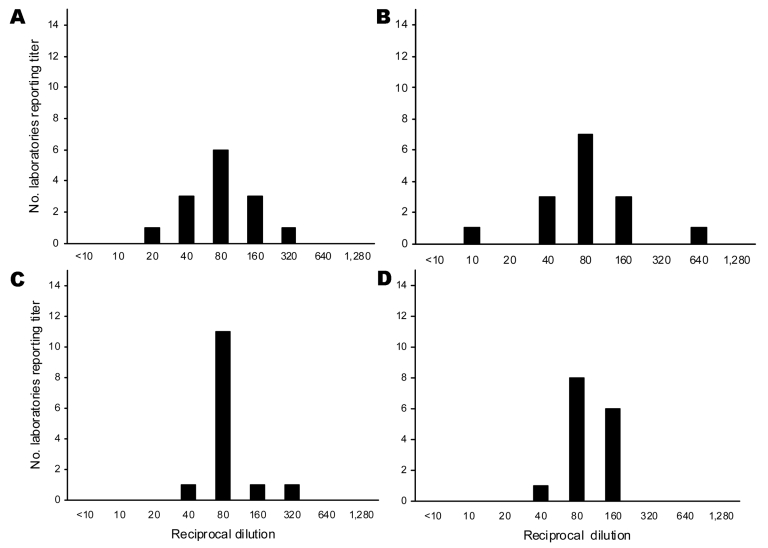
Range of hemagglutination-inhibition (HI) and neutralization titers to clade 1 homologous NIBRG-14 virus in postvaccination serum sample F: the number of laboratories reporting specific titer dilution of absolute titers and titers relative to 07/150. A) Absolute horse HI titers, B) absolute neutralization titers, C) titers relative to 07/150 horse HI titers, D) titers relative to 07/150 neutralization titers.

**Figure 3:**
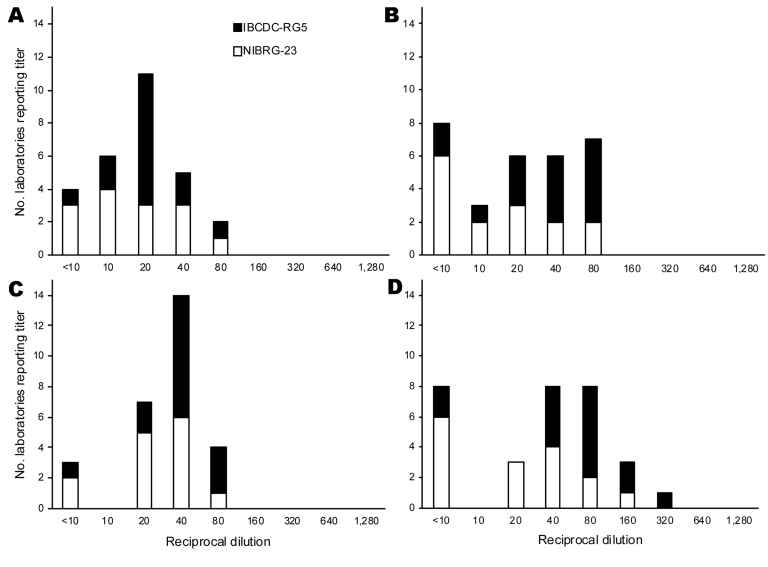
Range of hemagglutination-inhibition (HI) and neutralization titers to clade 2.2 and 2.3 heterologous NIBRG-23 and IBCDC-RG5 viruses in postvaccination serum sample F: the number of laboratories reporting specific titer dilution of absolute titers and titers relative to 07/150. A) Absolute horse HI titers, B) absolute neutralization titers, C) titers relative to 07/150 horse HI titers, D) titers relative to 07/150 neutralization titers.

To evaluate the ability of 07/150 to improve interlaboratory agreement, GMTs were expressed relative to 07/150 for each sample (Table 2) and then summarized for all samples (Table 3). For all serum, interlaboratory reproducibility improved significantly for NIBRG-14; the median %GCV for hHI decreased from 125% to 61% (p = 0.001) and for neutralization from 183% to 81% (p = 0.002, Wilcoxon signed-rank test). However, for clade 2 viruses, interlaboratory variation did not change significantly. Figures 2 and 3 display the range of absolute and relative 07/150 titers for each antigen in serum F (shown as an example of postvaccination serum with midpoint GMT and wide range of values).

For sheep antiserum, the interlaboratory variability was increased because some laboratories reported negative hHI titers, resulting in high %GCV when test serum samples were expressed relative to them (Table 3). However, when these laboratories were excluded from analysis, the interlaboratory variation for NIBRG-14 by hHI became comparable to that found for 07/150. Laboratory 5 reported negative hHI titers for serum P; when that laboratory was excluded from analysis, the range of %GCV by hHI improved from 689%–796% to 51%–71%. Laboratories 5, 6, and 12 reported negative hHI titers for serum O; when they were excluded, the range of %GCV improved from 306%–442% to 39%–113%. When neutralization titers were expressed relative to serum O, interlaboratory variation to NIBRG-14 was reduced, in contrast with serum P, for which interlaboratory variation by hHI or neutralization did not improve for any antigen, even when laboratory 5, which failed to detect antibody in this sample, was excluded.

### Relationship between hHI and Neutralization

Because a serum HI titer ≈40 is considered seroprotective (*2*)*,* the establishment of a consistent equivalence factor between an hHI titer of 40 and neutralization would be useful. However, the relationship of hHI and neutralization is dependent on the virus-serum-laboratory combination and cannot be generalized. Equivalence factors display large differences of 0.1–40.3 based on absolute titers and 0.3–6.3 based on titers relative to 07/150 for NIBRG-14 (Appendix Table 1).

### Serum K: False-positive Serum

Serum K was prevaccination serum from a person with detectable antibodies against influenza subtype H5 (by neutralization and hHI in the trial laboratory) but no known exposure to influenza subtype H5N1. Using NIBRG-14, NIBRG-23, and IBCDC-RG5 as test antigens, 13/14 (93%), 12/14 (86%), and 3/14 (21%) laboratories reported positive titers by both hHI and neutralization, respectively.

### Assay Operating Protocols

Thirteen laboratories supplied hHI protocols. Although similar (Appendix Table 2), they differed in some respects: pretest serum hemabsorption, erythrocyte suspension concentration (<1% vol/vol or >1% vol/vol), and time and temperature of erythrocyte-settling period (60 or >120 min, 4°C or room temperature). Although no relationship between protocol and intralaboratory reproducibility was found, laboratories that used lower erythrocyte concentrations or read plates at 4°C tended to report higher titers. Laboratories that performed pretest hemabsorption tended to report lower titers.

Thirteen laboratories supplied neutralization protocols (Appendix Table 3) that were grouped into 3 broad methods: use of cell suspension for virus infection with short incubation time to endpoint (<26 hours), use of cell suspension with long incubation (>3 days), and use of cell monolayer for infection with long incubation (>3 days). Although no parameters were clearly associated with reproducibility, laboratories that used monolayers tended to report lower titers than those that used cell suspensions, and those that used longer incubation times had more interlaboratory variation by more frequently reporting titers at either end of the range (i.e., highest or lowest) than laboratories that used shorter times. Expression of initial serum dilution varied among laboratories as dilution of test serum was calculated either before or after the addition of virus.

## Discussion

Having effective vaccines against influenza virus A (H5N1) is a public health priority. However, interlaboratory assay variation limits comparison of vaccine strategies without direct comparative studies. We compared the reproducibility of hHI and neutralization against a candidate standard. Overall, both assays were consistent, although neutralization displayed more intralaboratory variability than did hHI; 3 of 15 laboratories reported >2-fold differences in >25% of identical replicates.

Titers determined by neutralization were higher and had a greater range than those determined by hHI, which suggests that neutralization may be more sensitive, particularly with low-titered serum. However, for some prevaccination serum, e.g., sample N, 6 (40%) laboratories reported neutralization titers of 20–160 but negative hHI titers, which suggests nonspecific reactivity or that neutralization detects functionally different antibodies than HI. This finding is consistent with findings of seroprevalence surveys in which titers to influenza virus subtype H5N1 may be detected by neutralization but not HI or Western blot among some persons with no exposure to subtype H5N1 (*7*)*.* Sample K was from a person who had no known exposure but had detectable antibodies against H5. Most (93%) laboratories detected anti-H5 reactivity to NIBRG-14 by neutralization in this sample, but fewer (21%) detected antibodies to IBCDC-RG5. Studies suggest that antibodies against subtypes H1N1 and H3N2 detected by neutralization may be more strain specific than those detected by HI (*10*,*17*); however, we did not observe this difference.

Consistent with previous serologic comparisons (*10**,**11*)*,* interlaboratory variation was noted when absolute titers for the same serum samples were compared. Neutralization displayed more variability and had differences of 35-fold (%GCV 68%–232%) compared with differences of 16-fold (%GCV 22%–163%) for hHI. Although difficulty of measuring hHI values due to fragility of erythrocytes has been noted, the intralaboratory reproducibility of hHI appears better than that of seasonal HI (*10*,*11*). Both assays for subtype H5N1 compared favorably with those for subtype H3N2 evaluated previously, which found 128-fold (%GCV 138%–261%) and 724-fold differences (%GCV 256%–359%) with HI and neutralization, respectively (*10*)*,* and up to 32-fold differences (%GCV 90%–128%) with HI to human influenza subtypes H1N1, H3N2, and B viruses (*11*).

Although HI is straightforward, most laboratories preferred their own assays. Variable parameters that may influence hHI include pretest serum hemabsorption (lowers titers) and erythrocyte suspension (higher concentration lowers titers). Because no common neutralization protocols exist, laboratories have developed their own protocols, which creates potential for variability. Because operator inexperience may have influenced reproducibility of assays for subtype H3N2 (*10*), laboratories were selected for expertise in serologic testing for H5. Although most used microneutralization based on an assay described by the World Health Organization (*18*), protocols differed by starting dilution of serum; preparation of cells; and virus inoculation, incubation, and endpoint estimation. Laboratories that performed assays with virus infection of cell monolayers generally reported lower titers than those that used suspensions. Assays with long incubation times and non-ELISA endpoints (e.g., cytopathic activity) were associated with greater interlaboratory variation than ELISAs with shorter incubation times. A biostandard should reduce variation associated with assay differences because standardization of protocols may be limited by local availability of reagents.

Expression of the initial serum dilution, which clearly influences absolute titers, should be standardized. Although HI titers are typically expressed as the serum starting dilution before any addition of virus, calculation of starting dilutions for neutralization varies among laboratories. We propose that the calculated starting dilution for seasonal and avian influenza neutralization be expressed as serum dilution before the addition of virus (e.g., 5 µL serum in 45µL diluent plus 50 µL virus solution is considered as 1:10) as it is with HI.

Because the correlation between serum antibodies detected by hHI and protective efficacy against influenza subtype H5N1 is unclear, by default, immunogenicity criteria established for seasonal vaccines (*3*–*5*) are used for subtype H5N1 vaccines despite the lack of established immune correlates for neutralizing antibodies. Although hHI and neutralization titers correlate closely (*9*,*19*), this and other studies (*10*) find that the relationship depends on individual laboratory-antigen-serum combinations and cannot be generalized.

A potential limitation to this study is that 07/150 was derived from recipients of adjuvanted whole-virus vaccine but test serum samples were obtained from persons who received plain or adjuvanted split-product vaccines. Interlaboratory agreement improved when NIBRG-14, but not heterologous antigens, was used, which suggests that 07/150 is clade specific. Although no association between vaccine formulation and %GCV was noted in test serum, the quality and cross-reactivity of antibodies induced by whole-virus vaccine may differ from quality and cross-reactivity induced by alternative formulations including adjuvanted, subunit, or recombinant vaccines. To reduce potential variation in antibody isotypes, we obtained day-42 postvaccination samples when possible; however, the avidity of antibody to hemagglutinin or presence of antibody against denatured viral proteins after whole-virus vaccination (*20*) could influence the effectiveness of 07/150 against test serum. Differences among vaccine formulations should be examined, if possible, during evaluation of clade 2 standards; however, because production requires substantial donations of plasma, providing separate standards for specific vaccine formulations is impractical.

The overall reproducibility of sheep antiserum raised against clade 1 H5 hemagglutinin was poor; reported titers ranged widely. Because some laboratories failed to detect antibodies in sheep antiserum, the expression of relative titers did not reduce %GCV. When these laboratories were excluded from analysis, sheep serum improved interlaboratory agreement to NIBRG-14 by hHI but not by neutralization or for clade 2 antigens. This finding suggests that if assays can detect antibodies, sheep antiserum is a useful internal control; however, its role as an international standard is limited if some hHI assays appear unable to detect antibody titers. The reason for this discrepancy is unexplained because no clear association with assay method has been found. The antibody repertoire induced by cleaved hemagglutinin in Freund adjuvant in sheep antiserum will differ from that induced in humans by purified antigens. An alternative animal source and/or production method may be more reliable.

The World Health Organization Expert Committee on Biologic Standards has accepted 07/150 as an antibody standard for clade 1 H5 hemagglutinin and has assigned an arbitrary value of 1,000 IU. The assigned value of 1,000 IU is equivalent to an hHI titer of 140 (i.e., GMT to NIBRG-14 found across study laboratories), giving a seroprotective titer for 07/150 of ≈285 IU. For neutralization, a standard value of 1,000 IU for 07/150 would be equivalent to a neutralization GMT of 518. Because the relationship between hHI and neutralization is inconsistent and immune correlates are lacking, assigning a seroprotective level to neutralization is not possible. Useful information may be obtained by retesting serum from completed trials of clade 1 subtype H5N1 vaccine candidates against 07/150. Regulators will be required to discuss the interpretation of a standard before vaccine licensure for clinical use.

For standardizing serologic assays that use different influenza (H5N1) clades, a reliable animal serum source would be most convenient, but failure of some laboratories to detect antibody in sheep antiserum limits their use. The production of clade-specific standards for subtype H5 viruses will require human plasma donations, which can only be produced after initial clinical trials have been conducted. This requirement must be considered in future vaccine studies.

## Supplementary Material

Appendix Table 1 Equivalence factors for hHI titer of 40 and a neutralization titer based on absolute titers and titers relative to candidate antibody standard 07/150 for NIBRG14 test antigen in postvaccination serum*

Appendix Table 2 Comparison of variable parameters in hHI protocols*

Appendix Table 3 Comparison of variable parameters in virus neutralization assay protocols*

## References

[1] Zambon M. Laboratory diagnosis of influenza. In: Nicholson KG, Webster RG, Hay AJ, editors. Influenza. Oxford (UK): Blackwell Publishing; 1997. p. 123–56.

[2] Potter CW. Determinants of immunity to influenza infection in man. Br Med Bull. 1979;35:69–75. 367490 10.1093/oxfordjournals.bmb.a071545

[3] Wood JM, Newman RK, Ploss K. The use of correlates of immunity in European Union licensing of influenza vaccines. Dev Biol (Basel). 2003;115:9–16. 15088770

[4] Committee for Proprietary Medicinal Products. Note for guidance on harmonisation of requirements for influenza vaccines, March 12, 1997; CPMP/BWP/214/96 [cited 2009 May 5]. Available from http://www.emea.europa.eu/pdfs/human/bwp/021496en.pdf

[5] US Food and Drug Administration. Guidance for industry. Clinical data needed to support the licensure of pandemic influenza vaccines [cited 2009 May 5]. Available from http://www.fda.gov/cber/gdlns/panfluvac.pdf

[6] Nicholson KG, Colegate AE, Podda A, Stephenson I, Wood J, Ypma E, Safety and antigenicity of non-adjuvanted and MF59-adjuvanted influenza A/duck/Singapore/97 (H5N3) vaccine: randomised trial of two potential vaccines against H5N1. Lancet. 2001;357:1937–42. 10.1016/S0140-6736(00)05066-2 11425416

[7] Rowe T, Abernathy RA, Hu-Primmer J, Thompson XX, Lu X, Lim W, Detection of antibody to avian influenza (H5N1) virus in human sera by using a combination of serological assays. J Clin Microbiol. 1999;37:937–43. 10074505 10.1128/jcm.37.4.937-943.1999 PMC88628

[8] Stephenson I, Wood J, Nicholson K, Zambon MC. Sialic acid receptor specificity on erythrocytes affects detection of antibody to avian influenza haemagglutinin. J Med Virol. 2003;70:391–8. 10.1002/jmv.10408 12767002

[9] Stephenson I, Wood JM, Nicholson KG, Charlett A, Zambon MC. Detection of anti-H5 responses in human sera by HI using horse erythrocytes following MF59-adjuvanted influenza A/duck/Singapore/97 H5N3 vaccine. Virus Res. 2004;103:91–5. 10.1016/j.virusres.2004.02.019 15163495

[10] Stephenson I, Das RG, Wood JM, Katz JM. Comparison of neutralising antibody assays for detection of antibody to influenza A/H3N2 viruses: international collaborative study. Vaccine. 2007;25:4056–63. 10.1016/j.vaccine.2007.02.039 17412461

[11] Wood JM, Gaines-Das R, Taylor J, Chakraverty P. Comparison of influenza serological techniques by international collaborative study. Vaccine. 1994;12:167–75. 10.1016/0264-410X(94)90056-6 8147099

[12] Ferguson M, Heath A. Report of a collaborative study to calibrate the second international standard for parvovirus B19 antibody. Biologicals. 2004;32:207–12. 15572102 10.1016/j.biologicals.2004.09.004

[13] World Health Organization. Norms and standards: quality, safety and efficacy of medicines [cited 2009 May 5]. Available from http://www.who.int/medicines/areas/quality_safety/en

[14] The World Health Organization Influenza Program Surveillance Network. Evolution of H5N1 avian influenza viruses in Asia. Emerg Infect Dis. 2005;11:1515–22. 16318689 10.3201/eid1110.050644 PMC3366754

[15] World Health Organizaion. WHO Expert Committee on Biological Standardization [cited 2009 May 5]. Available from http://www.who.int/biologicals/expert_committee/TRS932CVR%20with%20full%20Texts.pdf

[16] World Health Organizaion. WHO biosafety risk assessment and guidelines for the production and quality control of human influenza pandemic vaccines [cited 2009 May 5]. Available from http://www.who.int/vaccine_research/diseases/influenza/ECBS_2005_Annex_5_Influenza.pdf

[17] Okuno Y, Tanaka K, Baba K, Maeda A, Kunita N, Ueda S. Rapid focus reduction neutralization test of influenza A and B viruses in microtiter system. J Clin Microbiol. 1990;28:1308–13. 2380359 10.1128/jcm.28.6.1308-1313.1990 PMC267925

[18] World Health Organization. WHO manual on animal influenza diagnosis and surveillance [cited 2009 May 5]. Available from http://www.who.int/vaccine_research/diseases/influenza/WHO_manual_on_animal-diagnosis_and_surveillance_2002_5.pdf

[19] Kayali G, Setterquist SF, Capuano AW, Myers K, Gill J, Gray G. Testing human sera for antibodies against avian influenza viruses: horse RBC hemagglutination inhibition vs. microneutralization assays. J Clin Virol. 2008;43:73–8. 10.1016/j.jcv.2008.04.013 18571465 PMC2574547

[20] Gulati U, Kumari K, Wu W, Keitel WA, Air GM. Amount and avidity of serum antibodies against native glycoproteins and denatured virus after repeated influenza whole-virus vaccination. Vaccine. 2005;23:1414–25. 10.1016/j.vaccine.2004.08.053 15661391

