# Supplementary material for: Reproducibility of Serologic Assays for Influenza Virus A (H5N1)
Source: Emerg Infect Dis. 2009 Aug;15(8):1250–9. doi: 10.3201/eid1508.081754 (PMC2815968; doi:10.3201/eid1508.081754)
Supplement: Appendix Table 1 — Equivalence factors for hHI titer of 40 and a neutralization titer based on absolute titers and titers relative to candidate antibody standard 07/150 for NIBRG14 test antigen in postvaccination serum* [file 08-1754_appT1-s1.pdf]

Publisher: CDC; Journal: Emerging Infectious Diseases
Article Type: Research; Volume: 15; Issue: 8; Year: 2009; Article ID: 08-1754
DOI: 10.3201/eid1508.081754; TOC Head: Research

Appendix Table 1. Equivalence factors for hHI titer of 40 and a neutralization titer based on absolute titers and titers relative to candidate antibody standard 07/150 for NIBRG14 test antigen in postvaccination serum*

Laboratory no.	Equivalence factors from HI to neutralization for (NIBRG-14)										
	Serum samples, absolute titers						Serum samples, titers relative to 07/150				
	07/150	E	F	G	H	I	E	F	G	H	I
1	4.8	6.0	2.4	4.2	5.2	3.0	5.0	2.0	3.5	4.4	2.5
2	40.3	10.1	5.0	32.0	12.7	8.0	1.0	0.5	3.2	1.3	0.8
3	4.5	2.3	2.8	2.8	2.7	2.0	2.0	2.5	2.5	2.4	1.8
4	2.1	1.6	1.1	1.5	1.1	1.1	3.0	2.1	2.9	2.2	2.2
5	1.1	0.3	0.3	0.3	0.3	0.2	1.0	1.1	0.9	1.2	0.7
6	4.0	0.8	0.6	0.6	0.8	0.5	0.8	0.6	0.6	0.8	0.5
7	1.3	0.6	0.8	1.0	0.8	0.3	2.0	2.5	3.2	2.5	1.0
8	2.0	0.9	0.6	1.1	0.6	0.5	1.9	1.2	2.2	1.1	1.0
9	1.0	0.1	0.1	0.3	0.1	0.1	0.4	0.4	1.3	0.5	0.3
10	6.0	2.0	1.6	2.5	1.3	3.9	1.3	1.1	1.7	0.9	2.6
12	0.8	0.3	0.3	0.4	0.4	0.3	1.6	1.6	2.0	2.0	1.3
13	8.0	12.7	5.0	6.3	12.7	4.0	6.3	2.5	3.2	6.3	2.0
14	12.7	5.0	2.5	8.0	2.5	4.0	1.6	0.8	2.5	0.8	1.3
15	7.3	1.1	1.1	1.7	1.4	3.1	0.6	0.6	0.9	0.8	1.7
Overall	3.6	1.4	1.1	1.8	1.3	1.1	1.6	1.2	1.9	1.5	1.2

*hHI, hemagglutination-inhibition assay using horse erythrocytes; neutralization, virus neutralization assay.
