# Supplementary material for: Reproducibility of Serologic Assays for Influenza Virus A (H5N1)
Source: Emerg Infect Dis. 2009 Aug;15(8):1250–9. doi: 10.3201/eid1508.081754 (PMC2815968; doi:10.3201/eid1508.081754)
Supplement: Appendix Table 2 — Comparison of variable parameters in hHI protocols* [file 08-1754_appT2-s2.pdf]

Appendix Table 2. Comparison of variable parameters in hHI protocols*

Parameter or variable	Most frequent variables used	Parameters used over all laboratories
Stock virus preparation		
Cell substrate for virus growth	10–11 day old embryonated eggs	10–11 day old embryonated eggs, MDCK
Conditions of virus growth	48 hr at 35°C	2–3 days at 34°C–37°C
Stock virus hemagglutination units titer	≥128	1:8 to 1:12800
Serum preparation		
Storage of serum after receipt	−20°C and 1 freeze thawing cycles	−20°C and 1–2 freeze-thaw cycles
Treatment of serum	3 RDE to 1 serum sample, overnight at 37°C, heat for 30 min at 56°C	3–5 parts RDE to 1 part serum, ± adsorption with horse erythrocytes
Sera diluent	Phosphate-buffered saline	Phosphate-buffered saline
Initial serum dilution	1:10	1:8 to 1:10
Serial dilution steps/volume	1:2 dilutions in 25-µL volume	1:2 dilutions in 25–50-µL volume
Range of serum dilutions	1:10 to 1:1,280	1:10 to 1:40,960
Cell preparation		
Cell type	Horse	Horse
Preparation of erythrocytes	Within 72 h of blood collection	Within 4 h to 4 wk of blood collection
Horse red cell diluent	Phosphate-buffered saline with 0.05% bovine serum albumin	+ 0.05%–5% BSA
Red cell suspension concentration	1% vol/vol	0.4%–2% vol/vol
Virus preparation		
Virus HA titration	4 hemagglutinin units with 1% horse erythrocytes	4–8 hemagglutinin units with 0.4%–1% horse erythrocytes
Volume of virus added	25 µL	25–50 µL
Virus/serum mix incubation conditions	30 min at room temperature	30–60 min at room temperature or 37°C
HI assay setup		
Total volume per well	100 µL	75–200 µL
Incubation conditions to HI endpoint	60 min at room temperature	60–130 min room temp or 4°C
Endpoint determination	Reciprocal of last well giving complete inhibition shown by streaming of RBC button	Reciprocal of last well giving complete inhibition as shown by streaming of erythrocyte button

*hHI, hemagglutination-inhibition assay using horse erythrocytes.
