# Supplementary material for: Reproducibility of Serologic Assays for Influenza Virus A (H5N1)
Source: Emerg Infect Dis. 2009 Aug;15(8):1250–9. doi: 10.3201/eid1508.081754 (PMC2815968; doi:10.3201/eid1508.081754)
Supplement: Appendix Table 3 — Comparison of variable parameters in virus neutralization assay protocols* [file 08-1754_appT3-s3.pdf]

Appendix Table 3. Comparison of variable parameters in virus neutralization assay protocols*

Parameter or variable	Most frequent variables used	Parameters used over all laboratories
Stock virus preparation		
Cell substrate for virus growth	10–11-day-old embryonated eggs, MDCK cells	
Conditions of virus growth	2 days at 35°C	2–4 days, 34 °C –37 °C
Stock virus infectivity and method of determination	≈10^6 $TCID_{50}$/mL, ≈1×10^9 PFU/mL	$10^{3.5-9}$ $TCID_{50}$/mL titrated by ELISA/cytopathic effect or PFU/ml
Serum preparation		
Storage of serum after receipt	−20°C and 1–2 freeze–thaw cycles	−20°C to −80°C and 1–3 thawing cycles
Pre-assay treatment of serum	Heat treatment, 56°C for 30 min	RDE then heat treat at 56°C for 30 min
Initial serum dilution	1:10 (10 µL in 90-µL diluent)	1:10-1:20 (5–40 µL in 45–360-µL diluent)
Sera diluent	DMEM	MEM, DMEM, PBS
Serial dilution steps	1:2 dilution steps	1:2
Range of serum dilutions	1:20 to 1:5,120	1:10 to 1:40,960
Virus preparation		
Virus concentration per well	100 $TCID_{50}$	11-100 $TCID_{50}$;0.2–0.3 PFU
Dilution of stock virus to achieve assay virus concentration	≥1:100	1:2 to 1:20,000
Volume of virus solution added	50 µL	50–200 µL
Virus diluent	DMEM	MEM, PBS
Virus/serum mix incubation	1 h at 37°C	1–2 at room temperature or 37°C
Calculated starting serum dilution	1:20	1:10 to 1:20
Cell preparation		
Preparation of cells and number of cells added	Cell suspension method	Cell suspension (1.5–5 × 10^4/well) Preformed monolayer (1.5–3 × 10^4/ well)
Cell type used	MDCK	MDCK
Assay diluent	DMEM with BSA, no trypsin	± added BSA and/or trypsin
Assay setup		
Total assay volume/well	200 µL	100–200 µL
Incubation time of assay to endpoint reading	<26 h	<26 h to ≥3 d
Incubation conditions	36°C in 5% CO_2	
Endpoint estimation		
Endpoint determination	Viral antigen detection by ELISA using antibodies against nucleoprotein	Detection of viral antigen by ELISA, cytopathic or hemagglutinin activity by light microscopy; cell viability by colorimetric staining
Endpoint calculation method	50% neutralization	100% neutralization No hemagglutinin activity

*$TCID_{50}$, 50% tissue culture infective dose; RDE, receptor-destroying enzyme; DMEM, Dulbecco modified Eagle medium; MEM, modified Eagle medium; PBS, phosphate-buffered saline; BSA, bovine serum albumin.
